# Generation of therapeutic protein variants with the human serum albumin binding capacity via site-specific fatty acid conjugation

**DOI:** 10.1038/s41598-017-18029-y

**Published:** 2017-12-21

**Authors:** Jinhwan Cho, Sung In Lim, Byung Seop Yang, Young S. Hahn, Inchan Kwon

**Affiliations:** 1 School of Materials Science and Engineering, Gwangju Institute of Science and Technology (GIST), Gwangju, 61005 Republic of Korea; 2 Department of Chemical Engineering, University of Virginia, Virginia, VA 22904 United States; 3 Department of Microbiology, University of Virginia, Virginia, VA 22904 United States

## Abstract

Extension of the serum half-life is an important issue in developing new therapeutic proteins and expanding applications of existing therapeutic proteins. Conjugation of fatty acid, a natural human serum albumin ligand, to a therapeutic protein/peptide was developed as a technique to extend the serum half-life *in vivo* by taking advantages of unusually long serum half-life of human serum albumin (HSA). However, for broad applications of fatty acid-conjugation, several issues should be addressed, including a poor solubility of fatty acid and a substantial loss in the therapeutic activity. Therefore, herein we systematically investigate the conditions and components in conjugation of fatty acid to a therapeutic protein resulting in the HSA binding capacity without compromising therapeutic activities. By examining the crystal structure and performing dye conjugation assay, two sites (W160 and D112) of urate oxidase (Uox), a model therapeutic protein, were selected as sites for fatty acid-conjugation. Combination of site-specific incorporation of a clickable p-azido-L-phenylalanine to Uox and strain-promoted azide-alkyne cycloaddition allowed the conjugation of fatty acid (palmitic acid analog) to Uox with the HSA binding capacity and retained enzyme activity. Deoxycholic acid, a strong detergent, greatly enhanced the conjugation yield likely due to the enhanced solubility of palmitic acid analog.

## Introduction

Use of therapeutic proteins to treat debilitating human diseases is exponentially growing and the worldwide market of therapeutic proteins has reached 167 billion dollars in 2010^1^. However, the clinical application based on therapeutic proteins is often limited. One major obstacle is their short serum half-life arising from renal filtration, proteolytic degradation, and pinocytosis. As a result, a severe clinical shortcoming resides in frequent injections, giving rise to poor patient compliance, high cost and thereby ultimately affecting patients’ quality of life. Therefore, the development of long-acting therapeutic proteins is urgent and beneficial for patients’ convenience and more cost-effective therapy^2,3^.

Conventionally, conjugation of poly(ethylene)glycol (PEG) to therapeutic proteins has been used to extend the serum half-life *in vivo* via several mechanisms, such as the reduction in renal filtration and protection from proteolysis. Some therapeutic proteins conjugated to PEG molecules were approved by the Food and Drug Agency (FDA) for disease treatment^2^. However, poor degradability and potential immunogenicity of PEG molecules^4^ drove the development of alternative strategies to extend the serum half-life of therapeutic proteins. The binding/conjugation of therapeutic proteins to HSA is highly effective in extending the serum half-life of therapeutic proteins^3,5^. Human serum albumin (HSA) has an inherently long serum half-life of 19 days in the human body^6^ due to the neonatal Fc receptor (FcRn)-mediated evasion from cellular degradation as well as the reduced renal filtration. Upon fluid-phase endocytosis, HSA is bound to FcRn at a low pH (6.0) of early endosomes and then recycled back into the blood instead of being degraded in lysosomes. HSA is then released into the bloodstream due to its low affinity to FcRn at pH 7^7,8^. HSA is known to be non-immunogenic, biocompatible, and degradable *in vivo* ^9^. Therefore, the HSA-binding/conjugation is considered as an attractive and promising strategy to extend therapeutic protein half-life and provides an alternative chemical modification to the conventional PEG conjugation.

**Figure 1:**
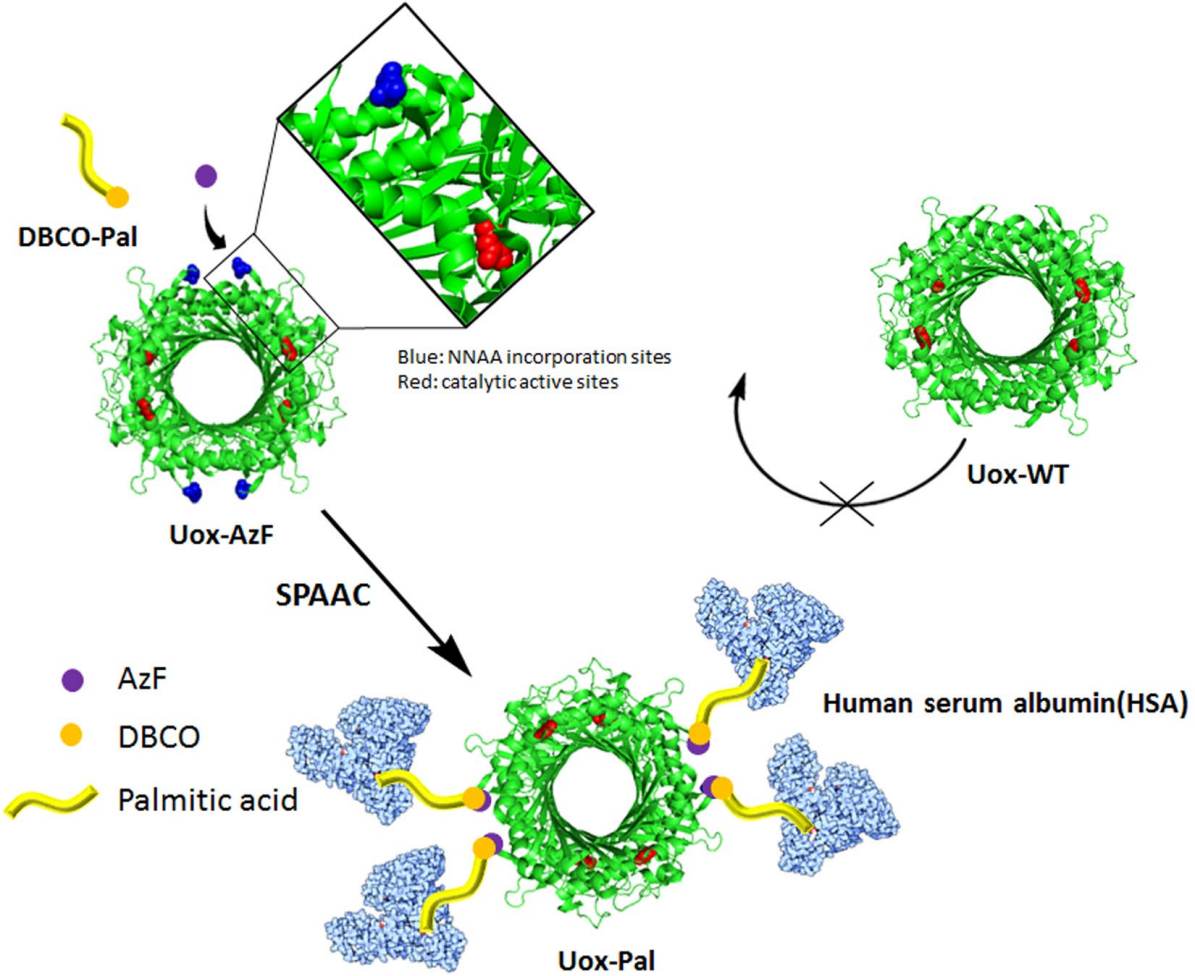
Scheme of site-specific palmitic acid-conjugation to urate oxidase (Uox) for acquired human serum albumin (HSA) binding capacity. A clickable non-natural amino acid, p-azido-L-phenylalanine (AzF) is introduced to a specific site of Uox. Palmitic acid analog containing dibenzocyclooctyne group (DBCO-Pal) is conjugated to AzF site of Uox via strain-promoted azide-alkyne cycloaddition (SPAAC). The palmitic acid-conjugated Uox (Uox-Pal) binds HSA, while wild-type Uox (Uox-WT) does not bind HSA. In the structure of Uox, AzF incorporation site and active site, which are marked in blue and red respectively, are distant from each other.

Herein, we investigated the conjugation of a natural HSA ligand, fatty acid, to a therapeutic protein to gain HSA binding affinity without compromising the therapeutic activity (Fig. 1). Compared to direct chemical conjugation of HSA to therapeutic proteins^5^, this approach has the advantages. First, a higher activity to mass ratio can be achieved^10^. Second, fatty acid is a lot cheaper than HSA. Given HSA has seven binding sites for saturated fatty acids^9,11,12^, the conjugation of a fatty acid to a drug is expected to facilitate the HSA binding in patients’ blood, resulting in the prolonged serum half-life *in vivo*. Recently, the conjugation of fatty acid to lysine residues of peptide drugs for diabetes treatment successfully extended the serum half-life *in vivo* ^13,14^. However, this strategy was not always suitable for therapeutic proteins since peptide drugs have few lysine residues but therapeutic proteins usually have many lysine residues. Therefore, fatty acid- or PEG-conjugation to multiple lysine residues of therapeutic proteins likely leads to heterogeneous mixtures of conjugates complicating downstream processes and compromising therapeutic activity. For instance, high level of random PEG-conjugation to human growth hormone results in a 1,500-fold reduction in the receptor binding affinity due to the PEG-conjugation at the receptor binding site, despite the 18-fold enhanced serum half-life^15^. Moreover, conjugation of fatty acid to interferon-α resulted in reduction in its antiviral activity by 80%^16^. Recently, we reported that site-specific fatty acid conjugation to superfolder green fluorescent protein (sfGFP) led to the prolonged serum half-life *in vivo* ^17^. The site-specific fatty acid conjugation was achieved by site-specific incorporation of a non-natural amino acid containing a bioorthogonal reaction handle to which fatty acid was conjugated via copper-catalyzed azide-alkyne cycloaddition (CuAAC). However, sfGFP is not a therapeutic protein. To our knowledge, site-specific conjugation of a fatty acid to a therapeutic protein was not yet reported. In this report, we optimized conditions for site-specific fatty acid conjugation to a model therapeutic protein, urate oxidase (Uox) and further investigated whether site-specific fatty acid conjugation can be achieved for a therapeutic protein obtaining in the HSA binding capacity without compromising the activity. The Uox (used in this study) originated from *Aspergillus flavus* consists of 301 amino acids and forms a homotetramer. Since Uox is a therapeutic enzyme catalyzing degradation of uric acid in the bloodstream, it is appropriate to investigate change in catalytic activity upon fatty acid-conjugation^18,19^. Recombinant Uox (marketed as Elitek in US) was approved by the FDA for the treatment of tumor lysis syndrome (TLS)^20^. TLS usually develops after treatment of leukemias and lymphomas^21^. Uox converts a water-insoluble uric acid into a water-soluble 5-hydroxyisourate, thereby facilitating easy excretion from kidney. To increase its half-life for long-term treatment, PEG was randomly attached to Uox^22^ for treatment of chronic gout (marketed as Krystexxa in US). Gout is a common inflammatory arthritis resulting from an elevated level of uric acid in the blood^23^. However, the generation of immunogenic responses against PEG have been problematic^24,25^. Therefore, an alternative strategy to extend serum half-life would be beneficial to clinical applications of Uox.

For successful site-specific fatty acid conjugation to a therapeutic protein via site-specific incorporation of a non-natural amino acid and click chemistry (Fig. 1), several factors need to be considered. First, conjugation reaction components/conditions often reduce therapeutic activities. For example, copper catalyst, a key component of CuAAC, has been reported to reduce catalytic activities of certain enzymes. In particular, in the presence of 1.0 mM CuSO_4_, the catalytic activity of Uox was significantly reduced (Supplementary Fig. 1). In order to address this issue, we employed strain-promoted azide-alkyne cycloaddition (SPAAC), copper-free click chemistry for fatty acid conjugation to Uox. Once p-azido-L-phenylalanine (AzF), a non-natural amino acid containing azido group, was site-specifically introduced to a specific site of Uox, a fatty acid derivative was conjugated to the azido group introduced to Uox via SPAAC. Recently, SPAAC was successfully used to conjugate HSA to Uox without compromising the catalytic activity^5^. Second, fatty acid conjugation efficiency varies on different conjugation sites. Taking advantage of site-specific introduction of AzF, we examined multiple conjugation sites to identify optimal conjugation sites. Third, linkers connecting fatty acid to Uox were investigated. Homo bi-functional and hetero bi-functional linkers were examined. Fourth, fatty acids or their derivatives are usually not soluble in aqueous solution. Such a poor solubility of fatty acid often leads to a poor conjugation yield. In order to address this issue, we investigated the effect of addition of a detergent deoxycholic acid (DCA) on fatty acid conjugation yield and Uox catalytic activity. Site-specific fatty acid-conjugation strategy developed here would be applied to the broad class of therapeutic proteins to generate their variants with the HSA binding capacity (Fig. 1).

## Materials and Methods

### Materials

p-Azido-L-phenylalanine (AzF, Supplementary Fig. 2a) was purchased from Chem-Impex International (Wood Dale, IL). Ni-nitrilotriacetate (Ni-NTA) agaroses and polypropylene columns were obtained from Qiagen (Valencia, CA). Vivaspin centrifugal concentrators with a 10-kDa molecular weight cut-off (MWCO) were purchased from Sartorius Corporation (Bohemia, NY). Sequencing-grade modified trypsin was obtained from Promega Corporation (Madison, WI). Membrane filters (0.45 μm pore size) were purchased from Advantec MFC Inc. (Dublin, CA). 15-Azidopentadecanoic acid (azide-Pal, Supplementary Fig. 2b) was obtained from Life Technologies Corporation (Gaithersburg, MD). Dibenzocyclooctyne (DBCO)-PEG4-DBCO (Supplementary Fig. 2c), DBCO-amine (Supplementary Fig. 2d), DBCO-PEG4-carboxyrhodamine (DBCO-Rho, Supplementary Fig. 2e) and DBCO-MB543 were purchased from Bioconjugate Technology Company (Scottsdale, AZ). Ziptip sample prep pipette tips with a C18 resin were obtained from Millipore Corporation (Billerica, MA). PD-10 desalting columns were obtained from GE Healthcare (Piscataway, NJ). Tris(hydroxymethyl)aminomethane (Tris), ampicillin, BCA protein assay kit, and Pierce® amine-binding maleic anhydride 96-Well plates were obtained from Thermo Fisher Scientific, Inc. (Rockford, lL). Plasmid Mini Extraction Kit was purchased from BIONEER Co. (Daejeon, South Korea). Sodium borate, sodium chloride, sodium phosphate monobasic, and sodium phosphate dibasic were obtained from DAEJUNG (Gyeonggi-do, Korea). All other chemicals were obtained from Sigma-Aldrich Corporation (St. Louis, MO), unless otherwise stated.

### Plasmid Construction for Expression of Wild-type Urate Oxidase and Variants

Preparation of the plasmid encoding recombinant urate oxidase gene originating from *Aspergillus flavus* (pQE80-Uox-WT) and the plasmid encoding a pair of engineered tyrosyl-tRNA/synthetase genes originally derived from *Methanococcus jannaschii* (pEVOL-pAzF) specific for AzF were described previously^5,26^. Preparation of the plasmid encoding the Uox variant gene containing an amber codon at W160 site (pQE80-Uox-160Amb) was also described previously^5^. The solvent accessibility prediction program (ASA-View)^27^ and the protein structure viewer program (PyMOL program)^28^ were used to select sites of Uox for AzF incorporation based upon the crystal structure of Uox (PDB ID: 1WS2). ASA-View program predicts a solvent accessibility value of each residue, normalized to value of 1^27^. Therefore, such an ASA value has a value between 0 and 1. An ASA value close to 1 means high solvent accessibility, indicating that the residue is easily accessible by solvent. For AzF incorporation, in addition to W160 site previously selected^5^, five new sites with relatively high solvent accessibility (ASA value > 0.5) and away from the catalytic active sites (E22, K23, D112, K138, and Q243) were selected. For generation of the five Uox variant genes containing an amber codon at the sites selected, site-directed mutagenesis was performed by PCR using pQE80-Uox-WT plasmid as a template to introduce an amber codon (UAG) at each site resulting in pQE80-Uox-Xamb plasmid (X is a residue to be replaced with an amber codon). Primers used in this study were obtained from Macrogen, Inc. (Seoul, South Korea). The pQE80-Uox-Xamb plasmids and their corresponding primers were as follows: 1) pQE80-Uox-E22amb (Uox-E22amb_F, 5′-gtacaaggtgcataaggactagaagactggcgtgcagacag-3′ Uox-E22amb_R, 5′-ctgtctgcacgccagtcttctagtccttatgcaccttgtac-3′), 2) pQE80-Uox-K23amb (Uox-K23amb_F, 5'-acaaggtgcataaggacgaatagactggcgtgca-3′; Uox-K23amb_R, 5′-tgcacgccagtctattcgtccttatgcaccttgt-3′), 3) pQE80-Uox-D112amb (Uox-D112amb_F, 5′-cccgcatggacatctagggaaagccacaccc-3′; Uox-D112amb_R, 5′-gggtgtggctttccctagatgtccatgcggg-3′), 4) pQE80- Uox-K138amb (Uox-K138amb_F, 5′-ggtcgacgtggtggagggatagggtatcgacatcaag-3′; Uox-K138amb_R, 5′-cttgatgtcgataccctatccctccaccacgtcgacc-3′),and 5) pQE80-Uox-Q243amb (Uox-Q243amb_F, 5′-tcctggccagacagtagctgattgagactgtg-3′; Uox-Q243amb_R, 5′-cacagtctcaatcagctactgtctggccagga-3′), For expression of wild-type Uox (Uox-WT), the pQE80-Uox-WT plasmid was transformed into Top10 *E. coli* cells, generating Top10[pQE80-Uox-WT] cells. Genomically engineered C321.ΔA.exp *E. coli* expression host cells^29^ were obtained from Addgene (ID: 49018) and then, transformed with pEVOL-pAzF and pQE80-Uox-Xamb plasmids, generating C321.ΔA.exp [pEVOL-pAzF] [pQE80-Uox-Xamb] cells.

### Site-Specific Incorporation of AzF into Uox

In order to express a Uox variant containing AzF at a specific site, the overnight culture of C321.ΔA.exp [pEVOL-pAzF] [pQE80-Uox-Xamb] cells were inoculated into 2 × YT medium containing ampicillin (100 μg/mL) and chloramphenicol (35 μg/mL) and incubated at 37 °C, 220 rpm. After 2.5 hr incubation, AzF (final concentration of 1 mM) in 0.2 M NaOH solution was added. After 10 min, 1 mM IPTG and 0.2% (w/v) L-(+)-arabinose were added to the culture in order to induce the expression of Uox variants. After induction, the cells were cultured for 5 hr, and then centrifuged at 5,000 rpm for 10 min to obtain cell pellets. Then, cell pellets were stored at −80°C until required for use. To purify the Uox variant containing AzF, the cell pellets were resuspended with the lysis buffer (50 mM sodium phosphate, 0.3 M NaCl, and 10 mM imidazole; pH 7.5) and then supplemented with 0.1 mg/mL lysozyme followed by incubation on ice for 15 min. The resuspended cell pellets were sonicated for 45 min (10 sec pulse/20 sec pause). After centrifugation at 13,000 rpm for 30 min, the supernatant was filtered using a membrane filter to remove impurities. Then, the supernatant was incubated with Ni-NTA agaroses at 15 °C, 220 rpm for 1 hr. The Ni-NTA agaroses and lysate mixture were poured into a column and washed by the washing buffer (50 mM sodium phosphate, 0.3 M NaCl, and 20 mM imidazole, pH 7.5). Uox variants were eluted by the elution buffer (50 mM sodium phosphate, 0.3 M NaCl, and 250 mM imidazole, pH 7.5) and then buffer-exchanged to a 20 mM sodium phosphate (pH 7.4) containing 0.2 M NaCl using a PD-10 column. Then, the purified Uox variant solution was concentrated to an appropriate protein concentration and stored at 4 °C until required for use. Expression and purification of Uox-WT were similarly performed except that TOP10 *E. coli* cells were used instead of C321.ΔA.exp cells and also AzF and L-(+)-arabinose were not used for protein expression.

The concentration of purified Uox-WT and Uox variants was measured using the Synergy™ microplate reader (BioTek, Winooski, VT). The molar extinction coefficient at 280 nm, ε_280_ (M^−1^ cm^−1^), of Uox-WT and Uox variants were calculated using the below equation (1)^30^.

1 $${\varepsilon }_{280}=(5500\times {n}_{Trp})+(1490\times {n}_{Tyr})+(125\times {n}_{disulfidebond})+(2620\times {n}_{AzF})$$

Briefly, the molar absorbance of Trp, Tyr, disulfide bond, and AzF are factors that determine the molar extinction coefficient of a target protein. The ε_280_ values of Uox-WT and Uox variants were calculated to be 53,520 M^−1^ cm^−1^ and 56,140 M^−1^ cm^−1^, respectively. The protein concentrations were calculated using the Beer–Lambert Law^31^.

### Mass Spectrometric Analysis

Uox-WT or Uox-160AzF in the storage buffer at 0.5 mg/mL were digested with trypsin at 37 °C for 2 hr, and then desalted on a ziptip C18 according to the manufacturer’s protocol. Eluted tryptic digests were mixed with 2,5-dihydroxybenzoic acid (DHB) matrix (20 mg/mL of DHB and 2mg/ml of L-(-)-fucose dissolved in 10% ethanol at 1: 1 (v/v), and then subjected to mass analyses by Microflex MALDI-TOF M/S (Bruker Corporation, Billerica, MA). Purified Uox-WT and six Uox variants (Uox-22AzF, Uox-23AzF, Uox-112AzF, Uox-138AzF, Uox-160AzF, and Uox-243AzF) were subjected to liquid chromatography-mass spectrometry (LC-MS) to determine their masses. LTQ-Orbitrap XL mass spectrometer coupled with electron spray ionization (Thermo Scientific, Waltham, MA) was used. The detailed procedure for LC-MS analysis was described previously^32^.

### Generation of Uox-Pal (Uox-160Pal and Uox-112Pal) Conjugate

In order to conjugate a palmitic acid to a Uox variant using a homo-bifunctional linker (DBCO-PEG4-DBCO), the purified Uox-160AzF was first reacted with the linker via SPAAC under the following condition: 40 μM Uox-160AzF and 120 μM DBCO-PEG4-DBCO in 20 mM HEPES/0.2 M NaCl (pH 8.2) containing 10% (v/v) DMSO at room temperature for 2 hr. Then, azide-Pal was directly added to the reaction mixture up to a final concentration of 200 μM, and reacted for 2 hr to generate Uox-160Pal. The reaction mixture was desalted using a PD-10 column.

In order to conjugate a palmitic acid to a Uox variant using a hetero-bifunctional linker (DBCO-amine), DBCO-amine and Palmitic acid N-hydroxysuccinimide ester (NHS-Pal, Supplementary Fig. 2f) at a ratio of 1: 5 were reacted in 20 mM Sodium phosphate/0.2 M NaCl (pH 7.4) containing 20% (v/v) DMSO at 37 °C for 12 hr generating DBCO-Palmitic acid (DBCO-Pal, Supplementary Fig. 2g). Then, unreacted NHS-Pal was quenched with Tris solution at 37 °C for 2 hr. DBCO-Pal was mixed with Uox-112AzF at a ratio of 2: 1 and reacted at room temperature for 2 hr, generating a Uox-112Pal conjugate. The sodium phosphate buffer containing 0.2 M NaCl and 1 % (w/v) DCA was prepared. In order to increase a conjugation yield, various concentrations of DCA were added to the reaction mixture. The unreacted DBCO-Pal was removed using a PD-10 desalting column. The isolated Uox-112Pal was stored at 4 °C until required for use.

Fluorescence dye conjugation assays were performed to determine the conjugation yield of a palmitic acid to Uox variants. Each Uox variant (Uox-22AzF, Uox-23AzF, Uox-112AzF, Uox-138AzF, Uox-160AzF, or Uox-243AzF) (50 μM) was reacted with 100 μM of DBCO-amine or dimethylsulfoxide (DMSO) as a control. After 2 hr reaction at room temperature, either DBCO-Rho or DBCO-MB543 (120 μM) was added and then reacted 2 hr at room temperature. The samples were subjected to SDS-PAGE analysis. Fluorescence image of the protein gel was taken by Bio-Rad ChemiDoc™ XRS+ (Hercules, CA) followed by Coomassie staining of the gel.

### *In Vitro* Enzymatic Activity and HSA Binding Assay

The concentration of Uox-Pal was determined by the BCA protein assay kit using the purified Uox-WT as a standard. In order to measure the enzymatic activity of Uox, 50 nM of Uox-WT, Uox variant, or Uox-Pal was incubated with 100 μM of uric acid in 200 μL uricase assay buffer containing 50 mM sodium borate (pH 9.5) and 0.2 M NaCl, respectively. The conversion of uric acid to 5-hydroxyisourate at 25 °C was determined by monitoring a reduction in uric acid absorbance at 293 nm using Synergy™ four multimode microplate reader (BioTek, Winooski, VT).

In order to compare HSA binding capacity of Uox-WT and Uox-Pal conjugates, HSA solution was incubated in a maleic anhydride-coated 96-well plate, and then quenched with an excess amount of Tris buffer. Either Uox-WT or Uox-Pal was put into the HSA-coated wells and incubated at room temperature for 30 min. After several washes with PBS buffer, Uox enzymatic activity in each well was measured as described above except that no additional Uox was added.

## Results and Discussions

### Site-Specific Incorporation of AzF into W160 site of Uox

It is well known that HSA has multiple binding sites for fatty acids including palmitic acid^9^. Furthermore, it was reported that fatty acid-conjugation to a peptide drug led to the enhanced serum half-life *in vivo* very likely via binding to HSA in the serum^13,14^. However, fatty acid-conjugation to a therapeutic protein often led to substantial reduction in the therapeutic activity due to several factors including steric hindrance of the critical sites by fatty acid. In this study, we investigated whether fatty acid can be conjugated to a permissive site of a therapeutic protein for the enhanced HSA binding capacity without compromising the therapeutic activity.

**Figure 2:**
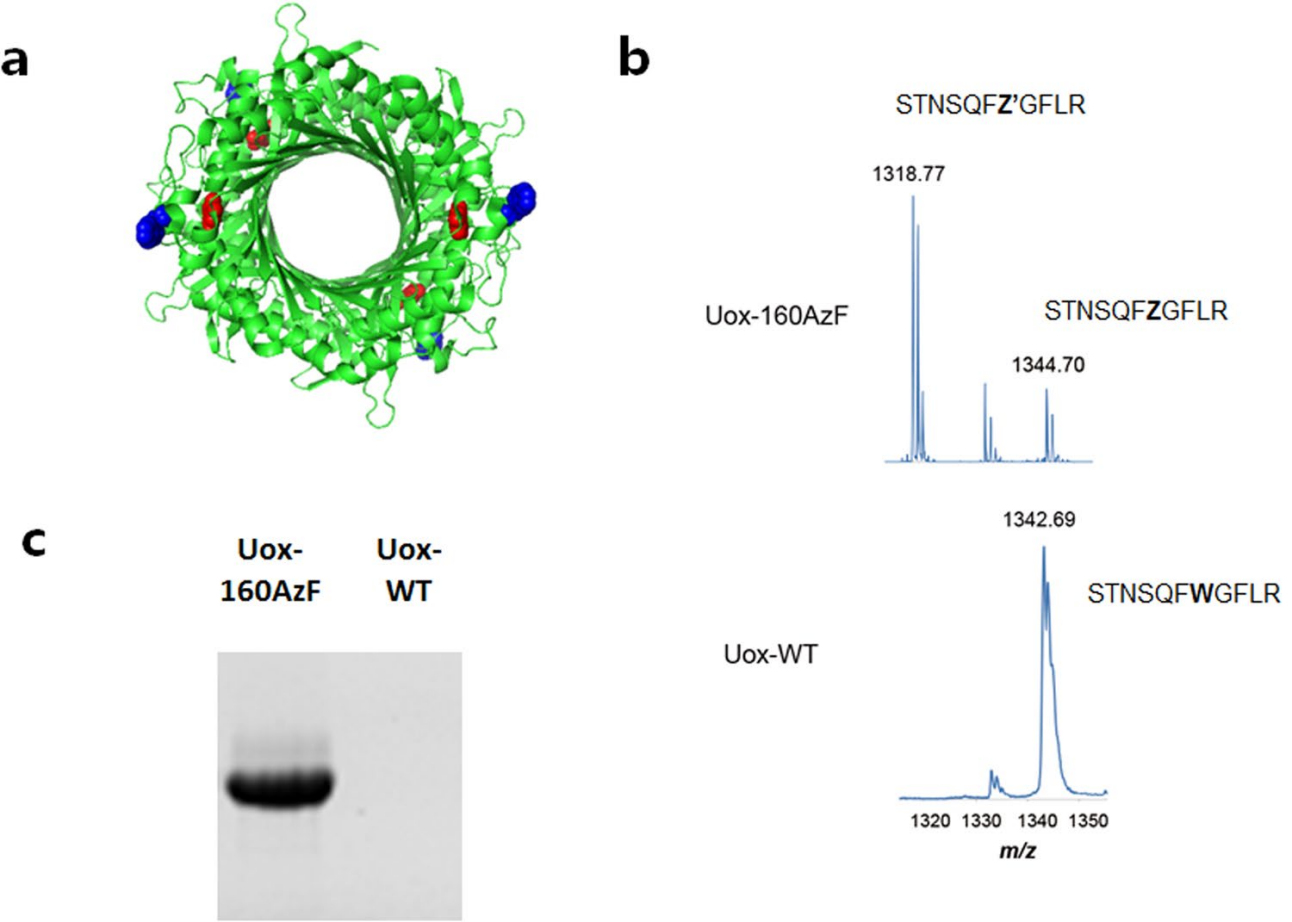
Site-specific incorporation of AzF into position 160 of Uox (**a**) Crystal structure of Uox (PDB ID: 1WS2) viewed by the PyMOL program. W160 and the active site are marked in blue and red, respectively. (**b**) MALDI-TOF spectra of Lys-C-digested fragments derived from the Uox-160AzF (top) and Uox-WT (bottom). (**c**) The fluorescence image of protein gel of Uox-160AzF and Uox-WT reacted with DBCO-Rho taken by Bio-Rad ChemiDoc™ XRS+ (excitation: 302 nm, emission: 510–610 nm).

First, we incorporated a clickable non-natural amino acid (AzF) into tryptophan residue at position 160 (W160) of Uox (Fig. 2a). In our previous study, AzF was introduced to two sites (W160 and W174) of Uox for HSA-conjugation^5^. However, the Uox variant containing AzF at position 160 (Uox-160AzF) was not yet extensively characterized. When the ASA-View program was used to evaluate the solvent accessibility, W160 site showed the solvent accessibility value greater than 0.4, indicating a good solvent accessibility. Moreover, substitution by AzF is unlikely to compromise the enzymatic activity of Uox, since it is away from the active site (Fig. 2a). In order to express Uox-160AzF, C321.ΔA.exp [pEVOL-pAzF] [pQE80-Uox-160amb] cells were cultured in the presence of AzF as described in Materials and Method section. Uox-160AzF was purified from the cell lysate via metal affinity chromatography using a hexa-histidine tag. Incorporation of AzF at the 160th position was confirmed by tryptic digest of Uox followed by MALDI/TOF mass spectrometry (Fig. 2b). For Uox-WT, the measured mass (*m*/*z*) of STNSQF**W**GFLR peptide (residues 154–164) was 1342.69, consistent with the expected mass (1342.69 *m*/*z*). For Uox-160AzF, the expected mass of STNSQF***Z***GFLR peptide (residues 154–164; ***Z*** = AzF) was 1344.70 (*m*/*z*). The measured mass of STNSQFZGFLR peptide was 1344.70 (*m*/*z*), clearly indicating incorporation of AzF at position 160. An additional peak appeared at a mass of 1318.77 (*m*/*z*). Considering that an azido group in AzF is known to be converted into an amine group upon laser irradiation^32^, the additional peak very likely indicated STNSQF***Z’***GFLR peptide (residues 154–164; ***Z’*** = amino-phenylalanine; expected mass of 1,318.67 *m*/*z*). To test the click chemistry reactivity and the bioorthogonality of the incorporated AzF, a fluorescent dye functionalized with DBCO (DBCO-Rho) was reacted with Uox-160AzF as well as wild-type Uox (Uox-WT) as a control (Fig. 2c). Only Uox-160AzF treated with the DBCO-Rho dye was fluorescent in in-gel analysis.

### Site-Specific Fatty Acid-Conjugation to Uox-160AzF Using a Homo-bifunctional Linker

**Figure 3:**
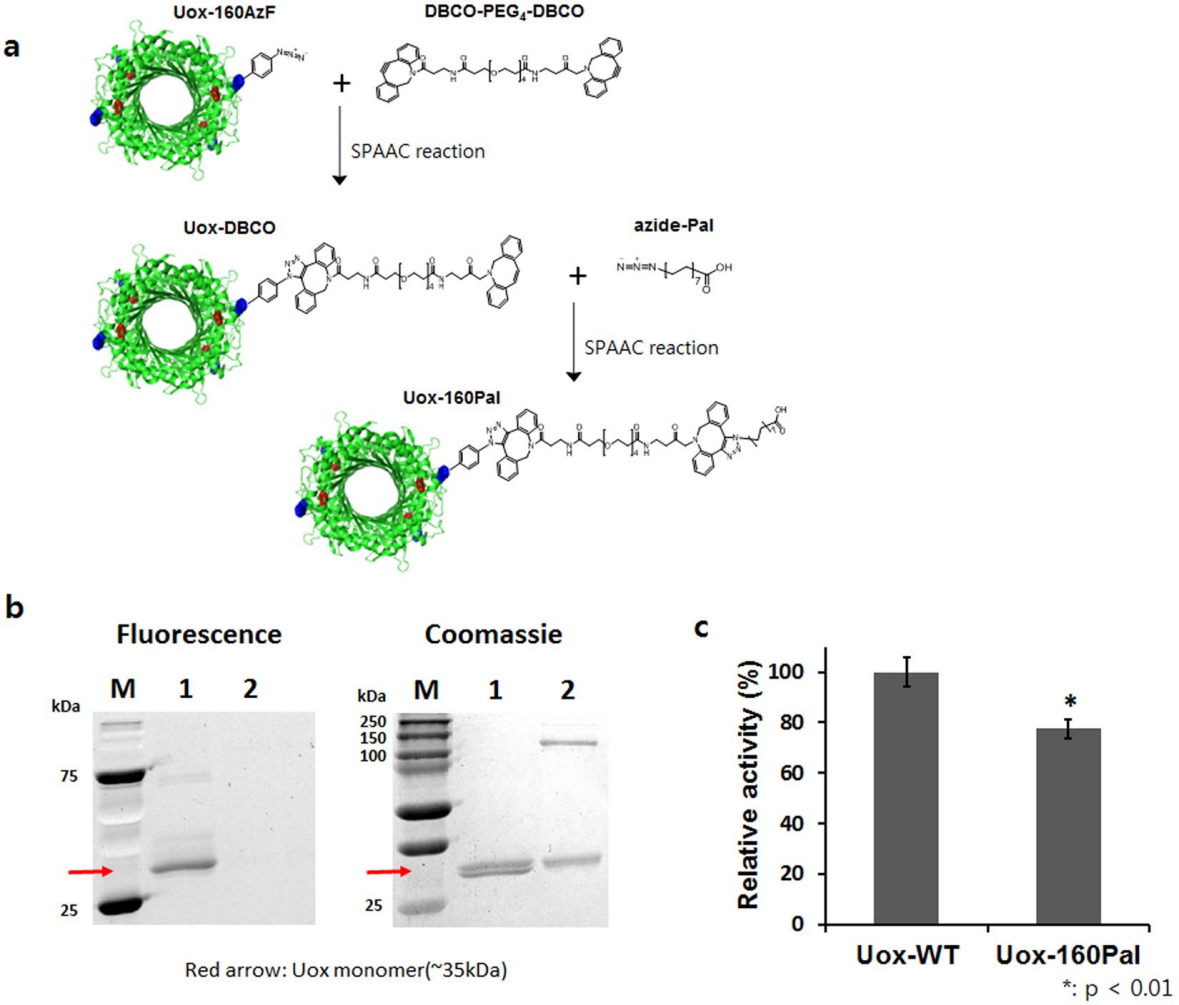
Construction scheme and characterization of palmitic acid-conjugated Uox-160AzF (**a**) Conjugation of Uox-160AzF and the palmitic acid analog containing an azide group (azide-Pal) via SPACC using a homo-bifunctional linker (DBCO-PEG4-DBCO) (**b**) The reaction mixture of Uox-160AzF and azide-Pal was further incubated with DBCO-Rho for fluorescence analysis. Fluorescence (Fluorescence Panel; excitation: 302 nm, emission: 510–610 nm) and Coomassie-stained image (Coomassie Panel) of protein gel of Uox-160AzF (lane 1) and Uox-160AzF reacted with DBCO-PEG4-DBCO linker (lane 2) taken by Bio-Rad ChemiDoc™ XRS+. M indicates a lane for molecular weight standards. (**c**) Enzymatic activity of Uox-160Pal relative to that of Uox-WT. Error bars represent standard deviations (n = 3). Enzymatic activity of Uox-160Pal was significantly reduced when compared to Uox-WT (two-tailed student’s t-test; *Indicates p < 0.01).

In order to conjugate a fatty acid to Uox-160AzF, a suitable crosslinker should be used. First, we used a homo-bifunctional linker containing DBCO group at both ends (DBCO-PEG4-DBCO) to conjugate azide-Pal to Uox-160AzF. Using this linker, we successfully conjugated (Rh)-coordinated organometallic electron mediator to a specific site of formate dehydrogenase enzyme via two-step SPAAC reactions^33^. Similarly, we first conjugated a DBCO-PEG4-DBCO linker to Uox-160AzF via SPAAC to generate a Uox-linker conjugate (Fig. 3a). The Uox-linker conjugate was incubated with an excess amount of azide-Pal to generate a Uox-Pal conjugate via SPAAC (Uox-160Pal) (Fig. 3a). However, Uox-160Pal conjugate had some issues. First, unwanted tetramer formation was observed. When Uox-160AzF was reacted with DBCO-Rho, two bands were observed in the Coomassie-stained protein gel (lane 1, Coomassie Panel, Fig. 3b). Since a tetrameric Uox is dissociated into monomers in the SDS-PAGE analysis, the bottom and top bands indicated unreacted monomeric Uox-160AzF and a monomeric Uox-dye conjugate, respectively. When Uox-160AzF was reacted with DBCO-PEG4-DBCO linker, one additional band was observed between 100- and 150-kDa molecular weight standards (lane 2, Coomassie Panel, Fig. 3b). Considering its molecular weight, the band was considered a tetrameric Uox. No fluorescence of the additional band was observed (lane 2, Fluorescence Panel, Fig. 3b), indicating that there was no free azide group in the tetramer. Therefore, we speculated that free DBCO group in the Uox-linker conjugate was further reacted to generate multimeric Uox species or unknown impurities in DBCO-PEG4-DBCO served as a crosslinker connecting Uox monomers/dimers. Second, the Uox-160Pal conjugate exhibited 20% lower enzymatic activity than that of Uox-WT (Fig. 3c). Third, conjugation yield of the DBCO-Rho dye to Uox-160AzF was about 50%. Therefore, we speculated that conjugation yield of the DBCO-PEG4-DBCO linker to Uox-160AzF was also moderate. In order to overcome several issues associated with azide-Pal conjugation to Uox-160AzF using DBCO-PEG4-DBCO linker, we further optimized the fatty acid-conjugation conditions.

### Screening of Conjugation Sites of Uox

**Figure 4:**
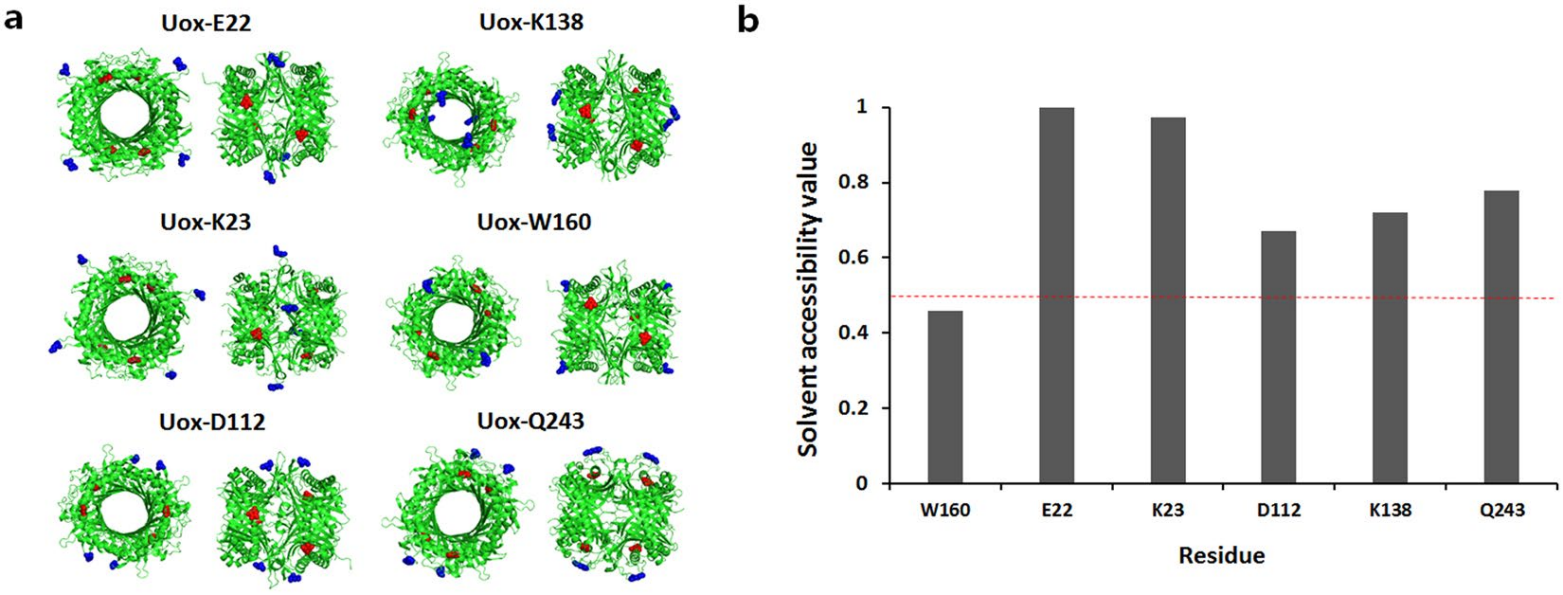
Candidate sites of Uox for site-specific incorporation of AzF. (**a**) Structures of six Uox variants (Uox-E22, Uox-K23, Uox-D112, Uox-K138, Uox-W160, and Uox-Q243). The crystal structure of Uox-WT (PDB ID: 1WS2) was viewed by the PyMOL program. The AzF incorporation site and active site are marked in blue and red, respectively. (**b**) Solvent accessibilities of site candidates for AzF incorporation, predicted by ASA-View Program. Red dot-line indicates the solvent accessibility value of 0.5.

In addition to W160 site, we decided to examine five additional sites of Uox (E22, K23, D112, K138, and Q243) for AzF incorporation and conjugation. In order not to interfere the catalytic property of Uox, we considered AzF incorporation sites away from the catalytic active site of Uox (Fig. 4a). Then, assuming the solvent accessibility is correlated to conjugation yield, the sites exhibiting higher solvent accessibility predicted by ASA-View Program were chosen for AzF incorporation and conjugation (Fig. 4b).

Five additional Uox variants containing AzF (Uox-22AzF, Uox-23AzF, Uox-112AzF, Uox-138AzF, and Uox-243AzF) were successfully expressed and purified as described in Materials and Method Section (Supplementary Fig. 3). In order to confirm the incorporation of AzF into each Uox variant, the purified five Uox variants (Uox-22AzF, Uox-23AzF, Uox-112AzF, Uox-138AzF, and Uox-243AzF) as well as Uox-WT were subjected to Q-TOF LC-MS analysis. The measured masses of Uox variants were almost identical to those of theoretical ones by less than 0.01% mass difference (Supplementary Fig. 4a and b).

**Figure 5:**
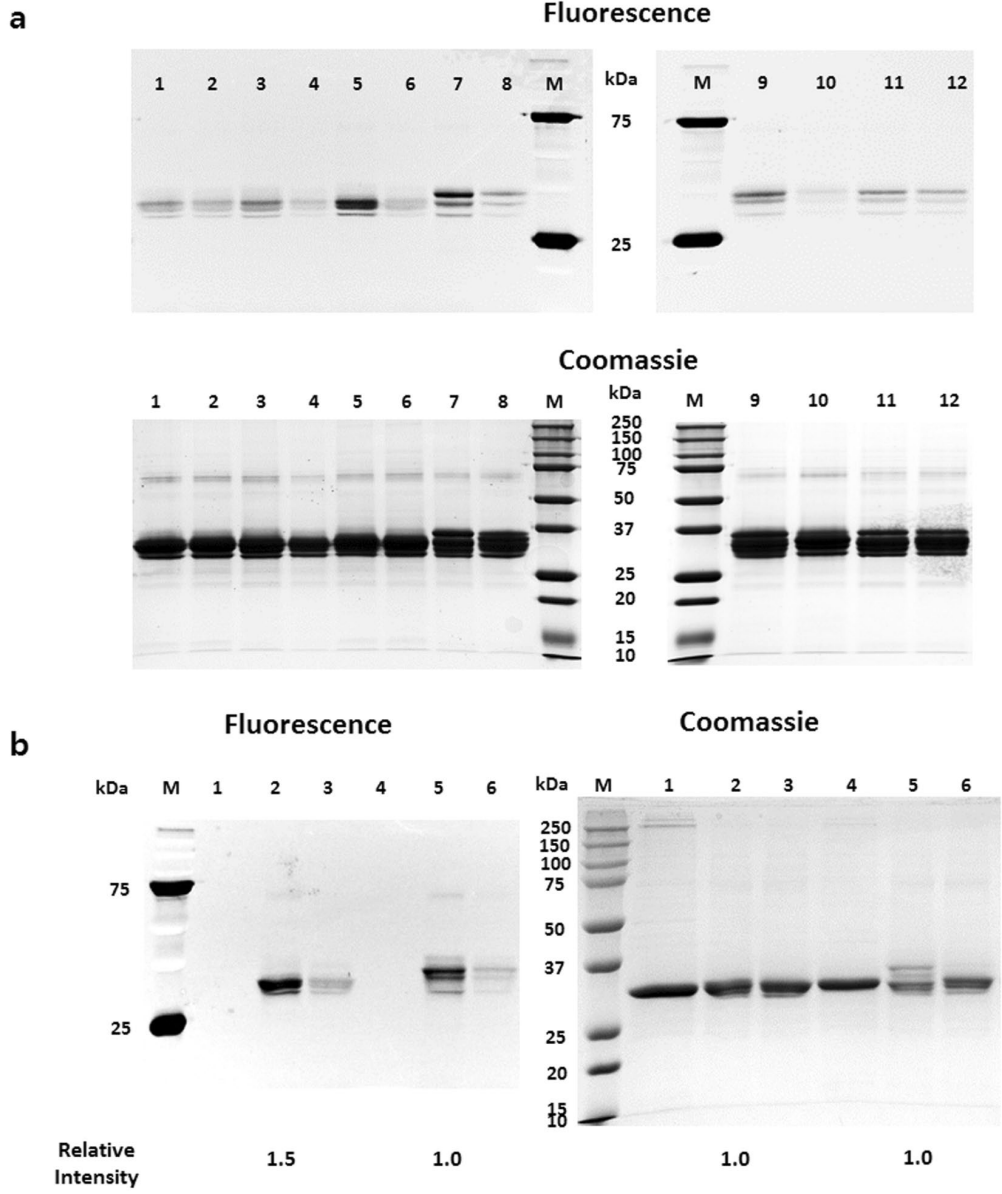
Comparison of fluorescence dye-conjugation yields of Uox variants. (**a**) Fluorescence (Fluorescence Panel; excitation: 302 nm, emission: 510–610 nm) and Coomassie-stained (Coomassie Panel) images of protein gels of Uox variant samples reacted with DBCO-Rho (Uox-22AzF (lane 1), Uox-23AzF (lane 3), Uox-112AzF (lane 5), Uox-138AzF (lane 7), Uox-160AzF (lane 9), and Uox-243AzF (lane 11)) and reacted with DBCO-amine followed by reaction with DBCO-Rho (Uox-22AzF (lane 2), Uox-23AzF (lane 4), Uox-112AzF (lane 6), Uox-138AzF (lane 8), Uox-160AzF (lane 10), and Uox-243AzF (lane 12)). (**b**) Fluorescence (Fluorescence Panel) and Coomassie-stained (Coomassie Panel) images of the protein gel of unreacted Uox variant (Uox-112AzF (lane 1) and Uox-160AzF (lane 4)), Uox variant reacted with DBCO-Rho (Uox-112AzF (lane 2) and Uox-160AzF (lane 5), and Uox variant reacted with DBCO-amine followed by reaction with DBCO-Rho (Uox-112AzF (lane 3) and Uox-160AzF (lane 6)). M indicates a lane for molecular weight standards. The images were taken by Bio-Rad ChemiDoc™ XRS+. The band intensities were analyzed by Image Lab software provided by Bio-Rad. Relative intensity values indicate the band intensities of Uox-112AzF samples relative to those of Uox-160AzF samples.

In order to choose a site with the highest conjugation yield, DBCO-Rho dye was conjugated to all Uox variants and fluorescence of the variants in the protein gels was compared (Fig. 5a). Among four Uox variants (Uox-22AzF, Uox-23AzF, Uox-112AzF, and Uox-138AzF), Uox-112AzF exhibited the highest intensity of fluorescence (lanes 1, 3, 5, and 7, Fluorescence Panel, Left gel, Fig. 5a). Among the remaining two Uox variants (Uox-160AzF and Uox-243AzF), Uox-160AzF exhibited a higher intensity of fluorescence than Uox-243AzF (lanes 9 and 11, Fluorescence Panel, Right gel, Fig. 5a). In order to determine a Uox variant with the best conjugation yield, the two Uox variants exhibiting the highest fluorescence from each gel (Uox-112AzF and Uox-160AzF) were subjected to DBCO-Rho dye conjugation analysis again (Fig. 5b). Upon conjugation to DBCO-Rho dye, the fluorescence intensity of the Uox-112AzF band was 50% higher than that of Uox-160AzF (lanes 2 and 5, Fluorescence Panel, Fig. 5b), while the amount of Uox-112AzF was comparable to that of Uox-160AzF (lanes 2 and 5, Coomassie Panel, Fig. 5b). In order to exclude the possibility of non-specific dye binding to Uox variants, Uox variants were reacted with DBCO-amine followed by the reaction with DBCO dyes, and then subjected to SDS-PAGE analysis for imaging. As expected, Uox-variants reacted with DBCO-amine showed very low fluorescence, indicating a minimal non-specific binding of DBCO dyes (lanes 2, 4, 6, 8, 10, and 12, Fluorescence Panel, Fig. 5a; lanes 3 and 6, Fluorescence Panel, Fig. 5b). The Dye-conjugation assay results revealed that D112 site exhibited the best conjugation yield among the six sites tested. D112 site had a relatively higher solvent accessibility than W160 site. However, since E22 and K23 sites with the highest solvent accessibility among the six sites tested did not exhibit good conjugation yield, we speculated that the solvent accessibility was not the only factor affecting conjugation yield and so there are other factors contributing to conjugation yield, such as local environment around a conjugation site.

### Site-Specific Conjugation of Fatty Acid to Uox-112AzF Using a Hetero-bifunctional Linker and a Detergent

**Figure 6:**
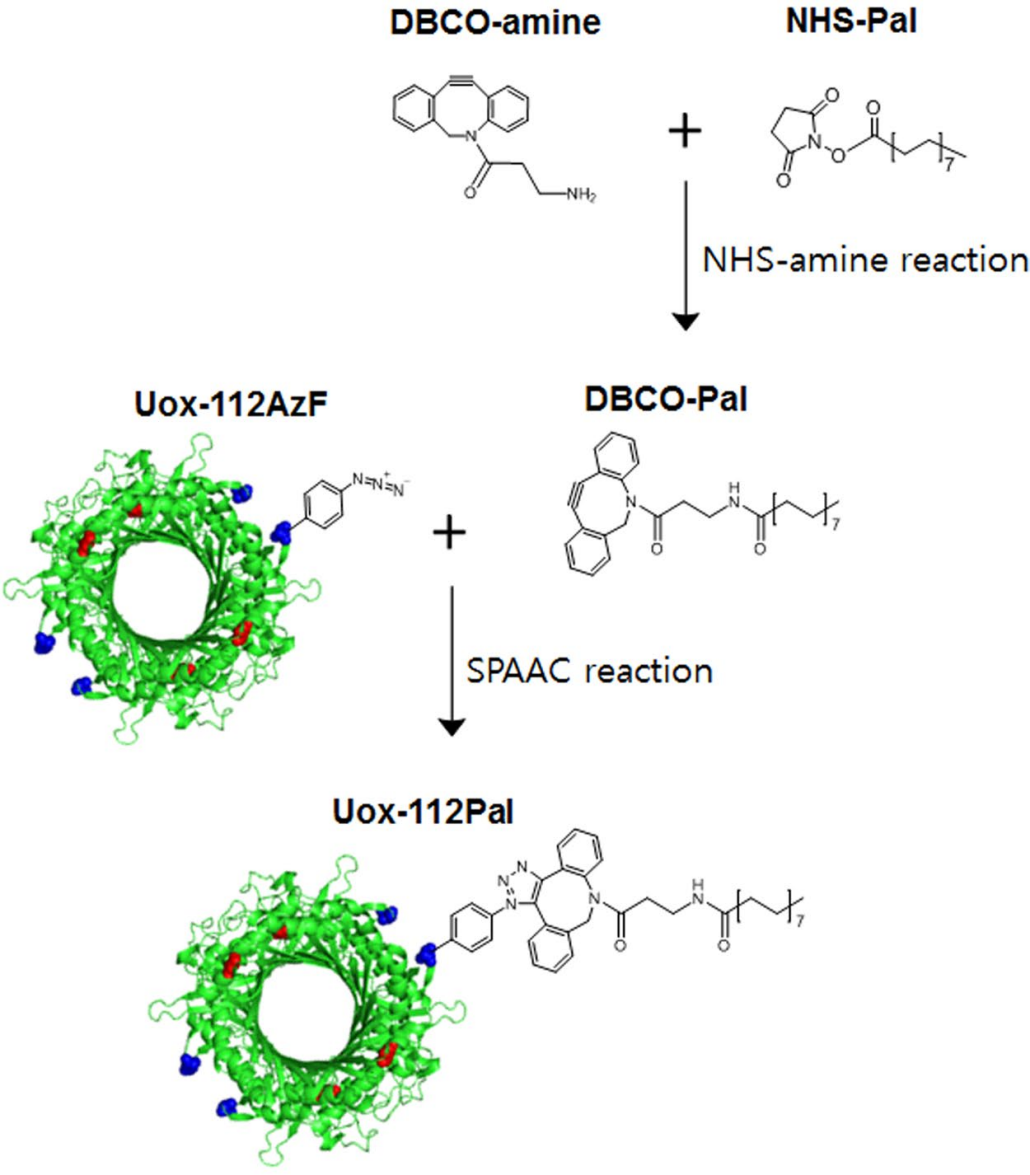
Construction scheme of palmitic acid-conjugated Uox-112AzF. Conjugation of a palmitic acid analog containing an NHS ester group (NHS-Pal) to an amine group of a hetero-bifunctional linker (DBCO-amine) to generate a DBCO-Pal conjugate. Then, conjugation of the DBCO-Pal to Uox-112AzF via SPACC.

**Figure 7:**
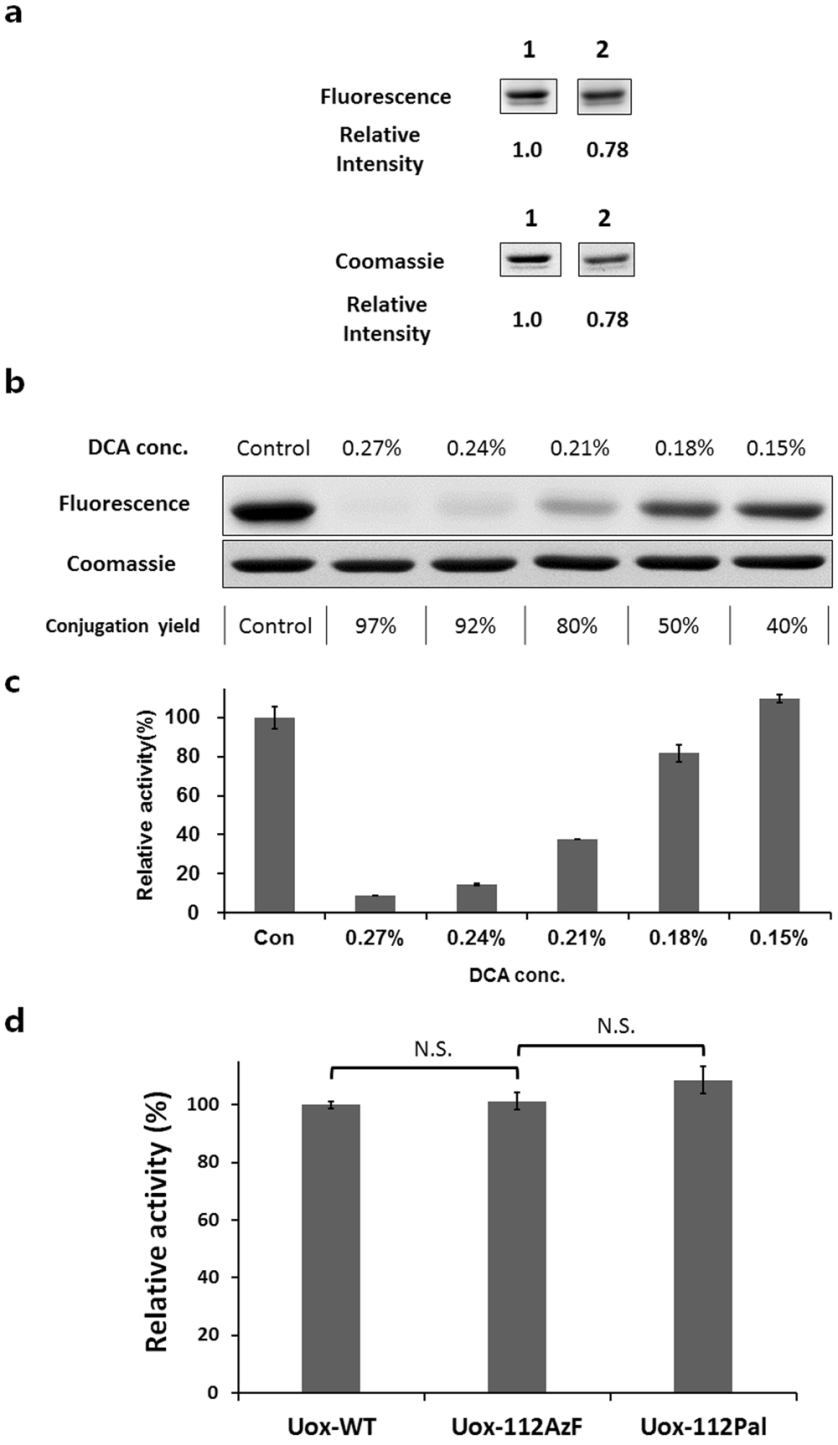
Effects of deoxycholic acid (DCA) addition on the conjugation yield of DBCO-Pal to Uox-112AzF. (**a**) Fluorescence and Coomassie-stained images of protein gels of the 50 μM Uox-112AzF samples incubated without (lane 1) or with (lane 2) 100 μM DBCO-Pal for 2 hr followed by reaction with 120 μM DBCO-MB543 fluorescence dye for 2 hr. Relative intensity values indicate the band intensities of Uox-112AzF samples incubated with DBCO-Pal relative to that of Uox-112AzF samples incubated without DBCO-Pal. These are cropped gel parts and the full-length gel images are shown in Fig. S5a. (**b**) Fluorescence and Coomassie-stained images of protein gels of the 50 μM Uox-112AzF samples reacted with 100 μM DBCO-Pal for 2 hr at varying concentrations of DCA (0, 0.15, 0.18, 0.21, 0.24, and 0.27 %) followed by reaction with 120 μM DBCO-MB543 fluorescence dye for 2 hr. Conjugation yields were calculated by the ratio of the fluorescence intensity of the sample prepared in the presence of DCA over that of the sample prepared in the absence of DCA. These are cropped gel parts and the full-length gel images are shown in Fig. S5b. (**c**) Enzymatic activity (100 μM uric acid) of the reaction mixture of Uox-112AzF (50 μM) and DBCO-Pal prepared at varying concentrations of DCA relative to that of the reaction mixture prepared in the absence of DCA. After the conjugation, the reaction mixture was desalted prior to enzymatic activity assays. Error bars represent standard deviations (n = 3). (**d**) Enzymatic activities of Uox-112AzF and Uox-Pal relative to that of Uox-WT (50 nM Uox variant, 100 μM uric acid, 30 min). Error bars represent standard deviations (n = 3). Enzymatic activity was not significantly different after incorporation of AzF and Pal conjugation. Enzymatic activities of Uox-112AzF and Uox-Pal were retained (two-tailed student’s t-test; N.S. indicates p > 0.01).

In order to avoid unwanted Uox multimer formation, we used a hetero-bifunctional linker to conjugate fatty acid to Uox-112AzF. As shown in Fig. 6, a hetero-bifunctional linker (DBCO-amine) was reacted with NHS-Pal to generate a DBCO-Pal conjugate. The DBCO-Pal conjugate was incubated with Uox-112AzF to generate Uox-112Pal via SPAAC. However, dye-conjugation inhibition assays showed that almost no conjugation of DBCO-Pal to Uox-112AzF was achieved probably due to poor water solubility (0.04 mg/L at 25 °C)^34^ of palmitic acid in the NHS-Pal/DBCO-Pal conjugates (Fig. 7a). In the dye-conjugation inhibition assays, a conjugation yield was calculated by comparing the specific fluorescence intensity of the sample prepared in the presence of DBCO-Pal over that of the sample prepared in the absence of DBCO-Pal. Addition of DBCO-Pal slightly diluted the concentration of the Uox-112AzF sample. Therefore, in the Coomassie-stained gel, the band intensity of Uox-112AzF sample incubated with DBCO-Pal (lane 2 in Coomassie Panel in Fig. 7a) was smaller than that of Uox-112AzF sample incubated without DBCO-Pal (lane 1 in Coomassie Panel in Fig. 7a). In Fluorescence Panel in Fig. 7a, the trends in fluorescence intensities of bands in lanes 1 and 2 were consistent with those in Coomassie Panel. Therefore, we concluded that the specific fluorescence intensities of Uox-112AzF samples incubated in the presence or absence of DBCO-Pal were comparable, demonstrating that there was almost no conjugation of DBCO-Pal to Uox-112AzF.

Palmitic acid is a fatty acid and does not dissolve in water. Furthermore, NHS-ester or DBCO group was connected to carboxyl group of palmitic acid, resulting in very poor water solubility. In order to enhance the water solubility of DBCO-Pal, we used deoxycholic acid (DCA) as a detergent in the reaction mixture. Since DCA was known to dissolve palmitic acid in aqueous solution^35–41^, we hypothesized that DCA can enhance the water solubility of DBCO-Pal too. As the concentration of DCA in the reaction mixture increased up to 0.27%, the fluorescence intensity decreased up to 3% but the conjugation yield of DBCO-Pal to Uox-112AzF increased up to 97%, based on the observation that the fluorescence intensity inversely correlated to the conjugation yield of DBCO-Pal to Uox-112AzF (Fig. 7b). However, as the concentration of DCA increased, the enzymatic activity decreased (Fig. 7c), indicating the inverse correlation between DCA concentration and enzymatic activity. DCA has a critical micelle concentration in a range of 0.083 to 0.249 % (w/v)^42^. Therefore, we speculated that the micelle formation of DCA led to efficient solubilization of DBCO-Pal but interfered with correct folding or tetramer formation of Uox. Therefore, we chose 0.15% as an optimal DCA concentration ensuring a good conjugation yield (~40%) without compromising the enzymatic activity. Finally, we compared the enzymatic activity of Uox-WT, Uox-112AzF, and Uox-112Pal. Uox-112Pal was obtained in the presence of 0.15% DCA as described above. No statistical difference (two-tailed student’s t-test; p > 0.01) was observed in the enzymatic activities among all three Uox samples (Fig. 7d). Uricase, the model therapeutic protein with multi-subunit, is a homotetramer. Since four azides were incorporated into a tetrameric uricase, maximum four fatty acids could be conjugated into a tetrameric uricase (Fig. 1). It was expected that Uox-112Pal has maximum four potential sites for albumin binding (Fig. 1). According to the conjugation yield of fatty acid to uricase, it was estimated that approximately 1.6 fatty acids were attached on a single tetrameric uricase.

### HSA Binding Assay

**Figure 8:**
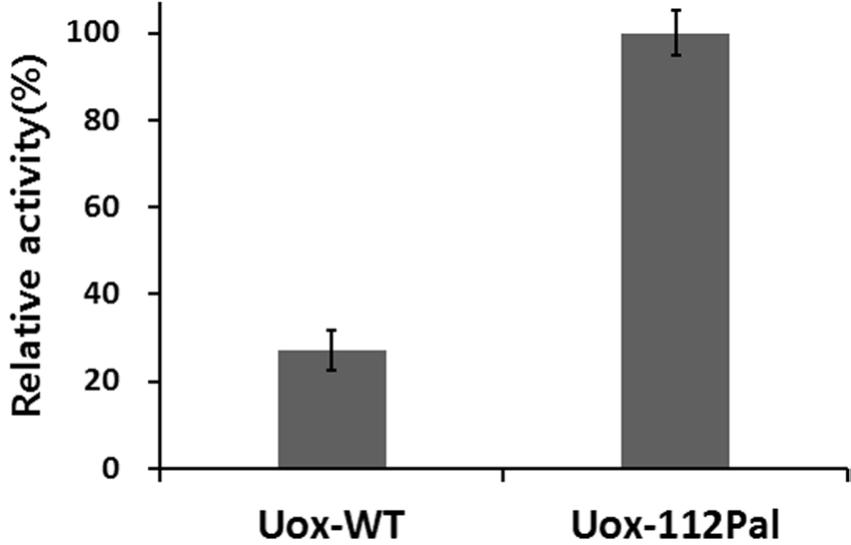
*In vitro* albumin binding affinity assay. Enzymatic activity of Uox-112Pal bound to the HSA-coated well relative to that of Uox-WT bound to the HSA-coated well measured at 100 μM uric acid for 30 min. Error bars represent standard deviations.

*In vitro* HSA binding assay was performed to determine whether Uox-112Pal had a HSA-binding capacity as described in Materials and Methods Section. As expected, Uox-112Pal exhibited 3.7-fold greater enzymatic activity than Uox-WT (Fig. 8). Since specific activity of Uox-112Pal was comparable to that of Uox-WT, we concluded that 3.7-fold more Uox-112Pal bound HSA-coated surface than Uox-WT. These results indicated that palmitic acid-conjugation to Uox-112AzF resulted in the effective HSA binding capacity. In the previous study, palmitic acid-conjugation to sfGFP led to 20-fold increase in the HSA binding capacity compared to unmodified sfGFP^17^. Relatively moderate increase in the HSA binding capacity of Uox-112Pal was attributed to the relatively lower conjugation yield (about 40%) compared with an almost complete conjugation yield in Pal-sfGFP generation. Therefore, if we could isolate the Pal-conjugated Uox-112AzF from the mixture of conjugate and unmodified Uox-112AzF, the difference in the HSA binding capacity between Uox-112Pal and Uox-WT would be greater.

## Conclusion

We have demonstrated that site-specific conjugation of palmitic acid analog to a permissive site of the Uox variant containing AzF resulted in the HSA binding capacity without compromising the enzymatic activity. In order to achieve this, we examined different types of click chemistries and linkers, various conjugation sites, and addition of a detergent. For copper-sensitive enzyme (Uox), SPAAC was required to maintain the enzymatic activity. Among the six sites with a good solvent accessibility and away from the active site, W160 and D112 sites exhibited the best conjugation yields. When a homo-bifunctional linker (DBCO-PEG4-DBCO) was used to connect azide-Pal to Uox-160AzF, a modest loss in the enzyme activity was observed, likely due to unwanted tetramer formation. Then, two-steps, NHS-amine and SPAAC reactions, were used to connect NHS-Pal to Uox-112AzF variant using a hetero-bifunctional linker (DBCO-amine). In order to solubilize the water-insoluble DBCO-Pal conjugate, a strong detergent (0.15% deoxycholic acid) was used. In the presence of 0.15% deoxycholic acid, the conjugation yield of DBCO-Pal to Uox-112AzF significantly increased up to 40% compared to almost no conjugation in the absence of deoxycholic acid. If a target therapeutic protein is not sensitive to copper, a higher conjugation yield of fatty acid to the therapeutic protein would be achieved via copper-catalyzed azide-alkyne cycloaddition (CuAAC) partly due to less steric hindrance by a small alkyne group compared with a bulky DBCO group^17^. Combined with the previous study of fatty acid-conjugation to a protein via CuAAC, the schemes and strategies described in this paper would serve as a template in order to site-specifically conjugate a fatty acid to a therapeutic protein without reducing the therapeutic activities. Ultimately, such a fatty acid-conjugation technique developed would be used to generate a therapeutic protein conjugate with the enhanced serum half-life *in vivo*.

## Electronic supplementary material

Supplementary Information
