# Supplementary material for: Generation of therapeutic protein variants with the human serum albumin binding capacity via site-specific fatty acid conjugation
Source: Sci Rep. 2017 Dec 21;7:18041. doi: 10.1038/s41598-017-18029-y (PMC5740134; doi:10.1038/s41598-017-18029-y)
Supplement: Supplementary file 1 — Supplementary Information [file 41598_2017_18029_MOESM1_ESM.pdf]

Generation of therapeutic protein variants with the human serum albumin binding capacity via site-specific fatty acid conjugation

Jinhwan Cho[1], Sung In Lim[2], Byung Seop Yang[1], Young S. Hahn[3], and Inchan Kwon*[,1, 2]

[1]School of Materials Science and Engineering, Gwangju Institute of Science and Technology (GIST), Gwangju 61005, Republic of Korea;

[2]Department of Chemical Engineering, University of Virginia, VA 22904, United States;

[3]Department of Microbiology, University of Virginia, VA 22904, United States

*To whom correspondence should be addressed. Street address: 123 Cheomdan-gwagiro, Buk-gu, Gwangju 61005, Republic of Korea; E-mail: inchan@gist.ac.kr; Tel.: +82-62-715-2312; Fax: +82-62-715-2304.

Supplementary Information

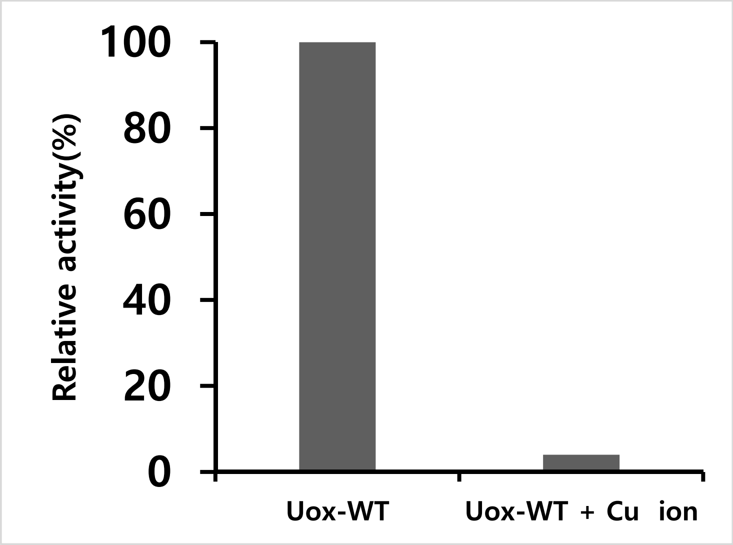

Fig. S1 Effect of copper ion on the enzymatic activity of Uox. In the presence of 1 mM copper ion, the enzymatic activity of 0.2 μM Uox-WT measured at 100 μM uric acid for 10 min was greatly reduced.

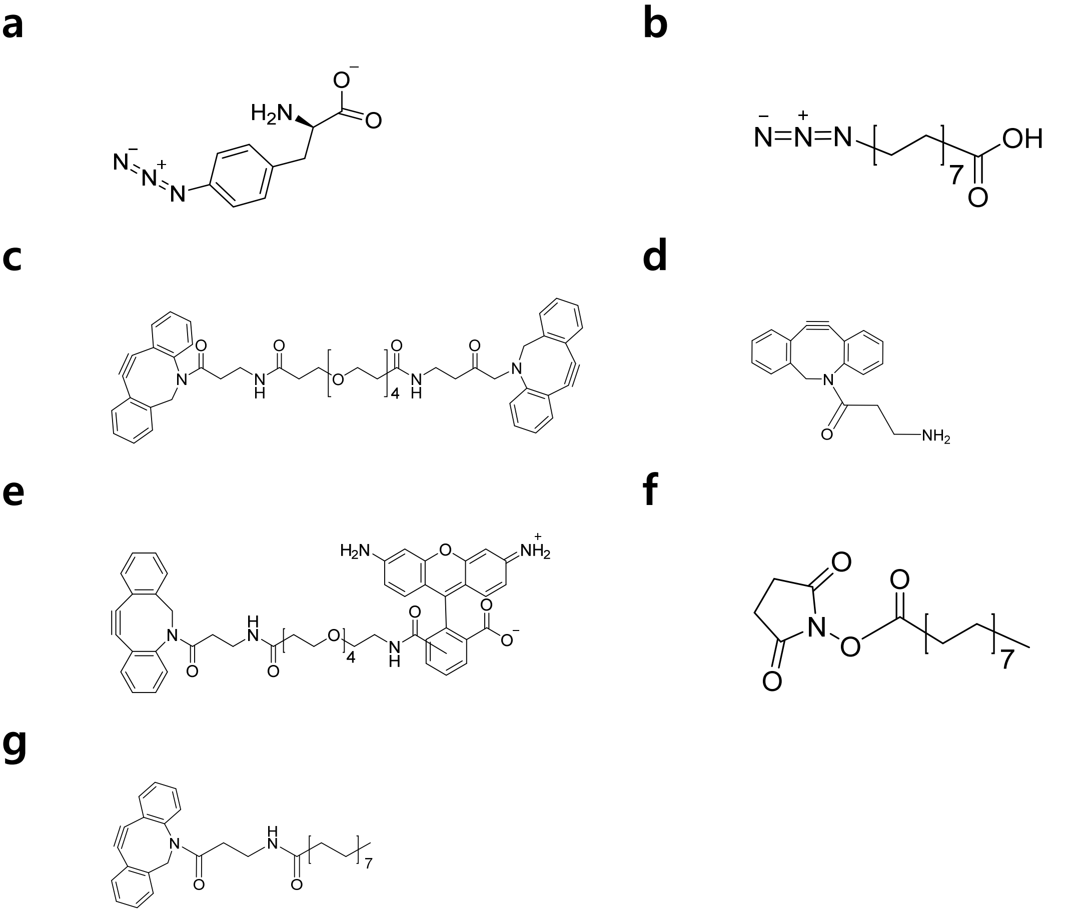

Fig. S2 Chemical structures of molecules described in this study. (a) p-azido-L-phenylalanine (AzF) (b) 15-Azidopentadecanoic acid (azide-Pal) (c) DBCO-PEG4-DBCO linker (d) DBCO-amine (e) DBCO-PEG4-carboxyrhodamine (DBCO-Rho) (f) Palmitic acid N-hydroxysuccinimide ester (NHS-Pal) (g) DBCO-palmitic acid (DBCO-Pal)

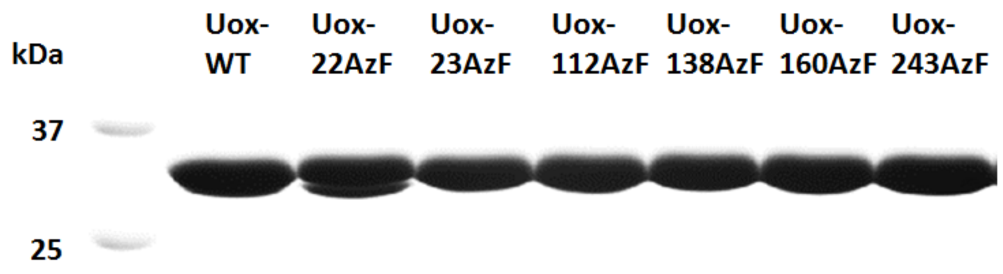

Fig. S3 Protein gel image of Coomassie-stained purified Uox variants (Uox-WT, Uox-22AzF, Uox-23AzF, Uox-112AzF, Uox-138AzF, Uox-160AzF, and Uox-243AzF).

a

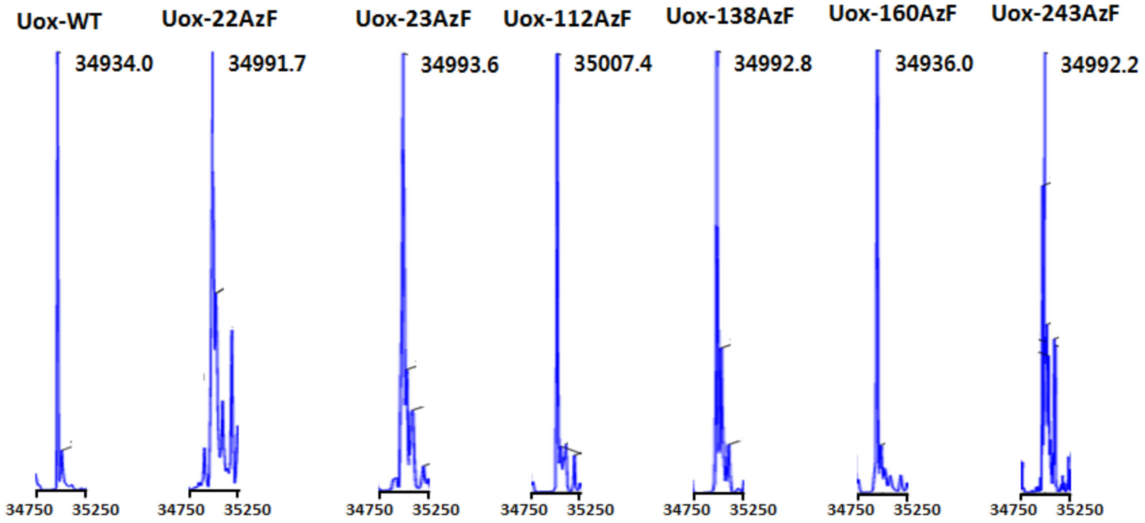

b

Uox variants	Theroetical mass (Da)	Experimental mass (Da)	Mass difference (%)
Uox-WT	34931.81	34934.0	0.006
Uox-22AzF	34990.88	34991.7	0.002
Uox-23AzF	34991.82	34993.6	0.005
Uox-112AzF	35004.91	35007.4	0.007
Uox-138AzF	34991.82	34992.8	0.003
Uox-160AzF	34933.78	34936.0	0.006
Uox-243AzF	34991.87	34992.2	0.001

Fig. S4 (a) LC-MS spectra of Uox variants (Uox-WT, Uox-22AzF, Uox-23AzF, Uox-112AzF, Uox-138AzF, Uox-160AzF, and Uox-243AzF). (b) The theoretical masses and experimental masses of Uox variants.

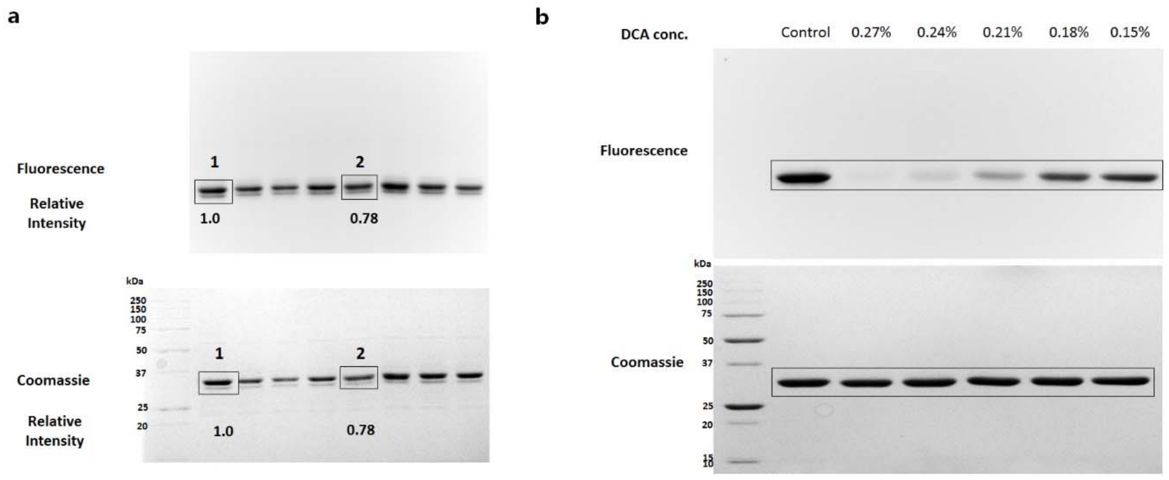

Fig. S5 Full-length protein gel images of which parts (marked with rectangles) were used to generate Fig. 7a (a) and Fig. 7b (b).
